# Community-Engaged Approaches for Improving the Inclusion of Diverse Communities in a Nutrition Clinical Trial

**DOI:** 10.3390/nu16213592

**Published:** 2024-10-23

**Authors:** Mopelola A. Adeyemo, Jessica Trinh, Darian Perez, Estabon Bozeman, Ejiro Ntekume, Jachael Gardner, Gail Thames, Tiffany Luong, Savanna L. Carson, Stefanie Vassar, Keith Norris, Zhaoping Li, Arleen F. Brown, Alejandra Casillas

**Affiliations:** 1 Department of Medicine, David Geffen School of Medicine, University of California Los Angeles, Los Angeles, CA 90095, USAjgardner@mednet.ucla.edu (J.G.); scarson@mednet.ucla.edu (S.L.C.);; 2 Mayo Clinic Alix School of Medicine Minnesota Campus, Rochester, MN 55905, USA; 3 Keck School of Medicine, University of Southern California, Los Angeles, CA 90033, USA; darianpe@usc.edu; 4 School of Public and Population Health, Boise State University, Boise, ID 83706, USA; 5 Department of Medicine, VA Greater Los Angeles Health Care System, Los Angeles, CA 90073, USA

**Keywords:** health disparities, cardiometabolic diseases, nutrition interventions

## Abstract

Background: Cardiometabolic disease (CMD) disproportionately affects African American/Black (AA) and Latino communities. CMD disparities are exacerbated by their underrepresentation in clinical trials for CMD treatments including nutritional interventions. The study aimed to (1) form a precision nutrition community consultant panel (PNCCP) representative of Latino and AA communities in Los Angeles to identify barriers and facilitators to recruitment and retention of diverse communities into nutrition clinical trials and (2) develop culturally informed strategies to improve trial diversity. Methods: A deliberative community engagement approach was used to form a PNCCP for the Nutrition for Precision Health (NPH) trial, part of the of the All of Us research initiative. The PNCCP included individuals that provide services for Latino and AA communities who met during 11 virtual sessions over 1 year. Discussion topics included enhancing recruitment and cultural acceptance of the NPH trial. We summarized CCP recommendations by theme using an inductive qualitative approach. Results: The PNCCP included 17 adults (35% AA, 47% Latino). Four thematic recommendations emerged: reducing structural barriers to recruitment, the need for recruitment materials to be culturally tailored and participant-centered, community-engaged trial recruitment, and making nutrition trial procedures inclusive and acceptable. We outlined the study response to feedback, including the constraints that limited implementation of suggestions. Conclusion: This study centers community voices regarding the recruitment and retention of AA and Latino communities into a nutrition clinical trial. It highlights the importance of community engagement early on in protocol development and maintaining flexibility to enhance inclusion of diverse communities in nutrition clinical trials.

## 1. Introduction

Cardiometabolic disease (CMD) encompasses a cluster of interrelated cardiovascular and metabolic disorders like hypertension, obesity, and diabetes that disproportionately affect morbidity and mortality in African American/Black (AA) and Latino populations when compared to non-Hispanic White peers [1]. These disparities may be influenced by multiple factors like epigenetics and indigenous or adaptive cultural practices but especially by the inequities in the distribution of social determinants of health such as food security, environment, and access to care [2].

The underrepresentation of minority populations in randomized clinical trials (RCTs) for CMD treatment and intervention studies weakens the evidence base for addressing health inequities [3,4]. For example, it is well established that modifiable CMDs (e.g., type 2 diabetes and hypertension) can be positively impacted by lifestyle modifications, like dietary changes [5,6,7,8]; however, many of these studies have been predominantly conducted among White European participants. As a result, it is unclear if these dietary changes may lead to the same cardiovascular benefits in underrepresented racial and ethnic minority groups (URM) in the US.

Overall, there has been a trend toward increased inclusion of URM in RCTs over the last 25 years. However, the percentage of AA and Latino participants included in RCTs was less than 7% in 2018, despite their making up one-third of the US population [9,10]. Recruitment and enrollment of underrepresented communities in clinical trials has been affected by a lack of awareness or understanding of clinical trials, medical and research mistrust, cultural and language barriers, discrimination and structural racism that impacts outreach and referrals, lack of research centers in community settings, collection of bio-samples in the setting of medical mistrust, and financial burdens of participating in clinical trials [3,11,12,13]. Addressing these barriers will be essential to including underrepresented communities in clinical trials and improving racial/ethnic disparities in CMD. The lack of proportional inclusion of these populations in studies leaves clinicians without generalizable data on which interventions are the most efficacious in helping AA and Latino adults prevent and treat CMD and related health determinants [13].

To inform recruitment, retention, accessibility, and acceptability of precision nutrition clinical trial procedures for AA and Latino participants, we used a Deliberative Community Engagement (DCE) approach (defined below) and formed a unique community advisory board [14,15,16,17,18]. This advisory board, the Precision Nutrition Community Consultant Panel (PNCCP), worked in partnership with the Nutrition for Precision Health (NPH) clinical trial for the National Institutes of Health (NIH) All of Us research initiative. This paper (1) describes the process of creating a PNCCP, (2) shares PNCCP perspectives on barriers and facilitators to recruitment, retention, accessibility, and acceptability, (3) summarizes PNCCP recommendations for culturally tailored recruitment strategies, and (4) describes the initial implementation of these recommended changes in the NPH trial at UCLA. This study aims to provide a framework for current and future clinical trial researchers to engage the community in improving representation of racial and ethnic minorities in nutrition clinical trials.

## 2. Materials and Methods

### 2.1. Study Context: NIH All of Us NPH Trial at UCLA

The Nutrition for Precision Health (NPH) study is the first ancillary study of the All of Us research program [19]. The NPH trial aims to develop algorithms that predict individual responses to foods and dietary patterns. The trial evaluates participants’ physiological responses to diet through 3 different modules that include consumption of (1) usual diet, (2) prescribed diet at home, and (3) prescribed diet in a domiciled setting, each for a period of 10–14 days. The overarching goal is to apply these trial findings for individualized dietary and nutrition recommendations that may prevent and/or treat chronic diseases.

Los Angeles County (LAC) is the most diverse and populous county in the US [10,20]: three-quarters of the county’s residents are non-White, and over one-third were born outside the US. AA and Latino residents are disproportionately impacted by cardiometabolic diseases such as obesity and diabetes [21,22], particularly for Black and Latino residents. LAC is thus an ideal setting in which to develop community-engaged solutions to increase representation in a clinical nutrition trial [10].

### 2.2. Deliberative Community Engagement

We used the social scientific DCE research approach to gather recommendations from the PNCCP. The approach involves recruiting community leaders representative of populations of interest to engage in education and information exchange with investigators and staff [23]. This is followed by structured discussions focused on understanding community values, social norms, obstacles, and perspectives around complex issues, leading to recommendations that may be considered for change in protocols and policies [12,24]. The research team has successfully used DCE to improve diversity in Los Angeles COVID-19 vaccine trials [12].

### 2.3. PNCCP Recruitment and Selection

Potential PNCCP candidates were identified in Fall 2022 before the NPH trial start date in November 2023 based on nominations from the UCLA Clinical Translational Research Institute’s (CTSI) Community Engagement & Research Program (CERP) community partners, staff, and faculty and members of the AA and Latino Community Action Boards from the National Heart, Lung and Blood Institute-funded UCLA Disparities Elimination through Coordinated Interventions to Prevent and Control Heart and Lung Disease Risk Study. Candidacy was based upon lived experience with CMD or stakeholders and leaders in community-based organizations that address nutrition or CMD (e.g., fitness, food pantries, church groups, community health workers, and promotors) serving Latino and AA community members.

Selection was made by the research team based on candidates’ racial and ethnic self-identification, occupation, employment, geographic residence, and community served to ensure that members represented the diversity of LAC. Invitations to participate in the PNCCP were sent to candidates, defining the role of the consultant, expectations, and compensation.

### 2.4. PNCCP Meeting Structure and Overview of Content 

Between September 2022 and October 2023, 11 PNCCP sessions (1.5–2 h) were conducted over Zoom. The virtual sessions allowed for geographic LAC accessibility. Panel members were compensated USD 300 per meeting.

To orient the panel to the first meeting, the research study team developed a lay-language briefing booklet that described the roles and goals of a PNCCP, an overview of nutrition and its impact on CMD, precision nutrition explanation, the clinical trial process, the importance of diversity in clinical trials, and protection of participants’ rights in clinical trials. The briefing booklet was emailed to the PNCCP before the first monthly virtual meeting.

PNCCP meetings included an overview of a study topic, content area, or study material review followed by a structured discussion. The year-long curriculum covered the following content areas: clinical trial design, study benefits and risks, nutrition science, precision health, racial and ethnic disparities in CMD, and clinical trial participation; see Table 1 for the PNCCP curriculum and structured discussion questions. The moderated discussion portion aimed to elicit PNCCP thoughts on participation and retention in the NPH clinical trial, potential strategies to mitigate barriers, and culturally informed recommendations for enhancing the acceptability of the NPH trial by soliciting community values, social norms, obstacles, and opportunities [24]. Our previous work using a Community Consultant Panel in a COVID vaccine trial provided insight into the prioritization of meeting discussion topics [12]. In line with the DCE approach, meeting discussion topics were prioritized to initially ensure the PNCCP had a shared understanding of the clinical trial process and the specific nutrition clinical trial that would be discussed. Additionally, initial meetings focused on nutrition education as well as racial and ethnic disparities in cardiometabolic disease, which are issues that, based on our experience working with the community in CERP, we knew were issues of importance to the communities that the panelists represented. Subsequent meeting topics were prioritized to coincide with the timeline of the clinical trial. National NPH materials were vetted by the PNCCP for tailoring and potential modification (e.g., study protocols, recruitment flyers, community outreach presentations, trial informational videos, and websites). Session facilitators included a CTSI CERP moderator and NPH study staff. Facilitators ensured equitable participation by panel members, encouraged panelists to share justifications for their views, clarified participants’ questions and concerns, and encouraged panelists to listen to and actively consider all perspectives [25,26,27]. Facilitators would intercede in panelist discussions as necessary to ensure all voices were heard. Facilitators would promote voices and opinions of those not heard to ensure their views were included in the discussion.

### 2.5. PNCCP Data Collection and Analysis

PNCCP meetings were digitally recorded and automatically transcribed. Two research team members reviewed each recording to refine the notes for each meeting. Transcriptions and meeting notes were independently reviewed by three members of the research team (SV, JT, and DP) to develop a set of recurring themes from the discussion meetings. The final definition for each theme, as well as example quotes, was developed by MA, with iterative feedback from the entire research team, until clear consensus was achieved—inductively arriving at the set of themes after group discussions [28]. In regard to theme summaries and definitions, the study team then revisited the transcripts and notes to identify additional quotes under these categories. The research team met weekly to review the panel feedback and discuss the feasibility of suggestions and strategies for operationalizing them. For these analyses, we summarize the PNCCP recommendations and whether and how they were implemented for the clinical trial.

## 3. Results

### 3.1. PNCCP Demographic Characteristics

The 17 PNCCP panelists are described in Table 2. Most participants identified as AA (35%) or Latino (47%). Gender was evenly distributed (47% male, 47% female), and one individual preferred not to answer. Most panel members’ highest degree completed was either a bachelor’s degree (29%) or a post-graduate degree (59%).

The majority of panel members had previous experience participating in a research study in collaboration with an academic institution (65%), with more than one-third of the PNCCP indicating that they had previously participated in a community consultant panel (41%). Most PNCCP members worked in health care, advocacy/policy, healthy equity, or education/training organizations in LAC.

### 3.2. PNCCP Recommendation Themes

We identified four overarching recommendations from the PNCCP: (1) reduce structural barriers to recruitment, (2) make recruitment materials culturally tailored and participant-centered, (3) conduct community-engaged trial recruitment, and (4) consider the acceptability of trial procedures for inclusivity of and acceptability by diverse populations. For each prevalent recommendation theme, we also outline how the study was able to respond to this feedback (including describing the real-world constraints that limited some of the modifications proposed by our community members).

(1) Reduce structural barriers to recruitment.

The panel discussed structural barriers to clinical trial participation, including those related to clinical trials awareness and accessibility (Table 3).

*Clinical trial understanding impacted by history of racism*. The PNCCP identified a lack of experience and knowledge of the clinical trial process as a barrier to participation. The PNCCP further commented that this lack of understanding augments the historical medical mistrust within AA and Latino communities. One panelist commented, “*[there’s] a lot of misunderstanding about clinical trials. [So] some type of…presentations by trusted individuals …would help*”.

*Clinical trial accessibility*. The panel members discussed the ability to secure care for a child or elderly family member as a potential barrier to participation. The panel recommended expanding study hours to include weeknights and weekends for those unable to take time off work.

*Geographic and financial compensation considerations*. The PNCCP discussed the financial impact that participating in a clinical trial might have on individuals with economic instability. The panel expressed concern that the amount of compensation for participation in this multi-site study was inadequate given the cost of living and transportation in the LAC region compared to other study sites, as well as the time needed to commute to the UCLA trial site. The PNCCP also expressed concern that the amount of compensation may impact public benefits and taxes. A PNCCP member stated, “*something to think about is that … [the compensation] could potentially raise people’s income… to a level where they are no longer eligible for other benefits*”.

(2) Need for recruitment materials to be culturally tailored and participant-centered

The PNCCP reviewed national All of Us and NPH materials including infographics and videos and provided feedback to improve the acceptability and cultural sensitivity of the recruitment materials.

*Reduce scientific jargon*. PNCCP noted that scientific jargon on recruitment materials made understanding the study purpose, expectations, and benefits difficult. They recommended replacing scientific jargon with lay language, including replacing the term “precision nutrition” with “personalized nutrition” to improve understanding.

*Simplify messaging*. The panel highlighted that the length of text in recruitment materials made it challenging to keep the attention of a potential participant.

*Increase representation in images*. Additionally, the panel members noted that the imagery did not reflect a diverse population. For example, one panel member noted, “*It was really hard to find [an image] that I identify with, so I prefer a different flyer for different communities*”.

*Improve the diversity of contact methods*. The PNCCP noted a lack of variation in contact methods on the flyers, which was limited to a website and QR code. They also indicated that the ease with which participants can reach the research team with concerns and questions can serve as a facilitator to recruitment. They noted that limiting methods of contacting the research team to the study website may create a barrier to participation for those with poor digital literacy or access to the internet.

*Advertise and highlight study participation benefits*. PNCCP members recommended sharing the amount of study compensation and highlighting study benefits on recruitment flyers. The panelists discussed that the study had potentially significant societal benefits that were not highlighted in recruitment materials, which may serve as a facilitator to recruitment, with one panelist suggesting that, “*another ‘selling point’ is that the research could be the first study of an entire society, the results of which could improve the health of the entire nation, including all races, genders, ethnicities, etc.*”.

(3) Community-Engaged Trial Recruitment

The PNCCP provided insight into methods to optimize community engagement to improve trust in the relationship between the community and research team. *Community partner outreach*. The PNCCP endorsed the importance of leveraging existing community networks for recruitment. They suggested that partnering with community-based organizations that have already established trust with diverse community members may facilitate recruitment. A PNCCP member stated, “*it would be more effective…[recruiting] through trusted organizations versus recruiting through medical professionals because of …medical mistrust … Churches and other communities’ organizations already have a long-standing relationship with people in the community who are trusted*”. Another panelist discussed the importance of informed outreach from a community centered perspective, stating, “*Communication is key, and having leadership and having people disseminate this information from the perspective of the good that it’s going to do for a long time …There’s a lot the powerful information that will come from this*”.

*Utilize networking*. They also discussed encouraging individuals who have participated in the study to invite friends and family to participate in the study, as the experiences of trusted friends and family members would be valued in diverse communities.

*Improve study staff/researcher representation*. The PNCCP emphasized the importance of having a diverse research team that is reflective of the same communities at outreach events. The panel also identified the important role the research team’s rapport with participants plays in recruiting and retaining diverse participants. They discussed that due to a history of medical mistrust amongst underrepresented groups, having individuals in the research staff who reflected their community at outreach events and study visits may improve the level of trust. A panelist stated, “*Having researchers who come from the same communities they serve often goes a long way toward building trust*”.

(4) Making Nutrition Trial Procedures more inclusive and acceptable

The panelists discussed aspects of the study protocol that could be barriers to participation for diverse communities.

*Simplify consent form*. After reviewing the consent form, the PNCCP noted that the use of scientific jargon made understanding scientific procedures and participants’ expectations regarding time commitment and processes difficult to understand. Thus, they recommended limiting scientific jargon, using images to describe procedures, and thoroughly explaining the participants’ expectations regarding time commitment and processes to improve potential participants’ understanding of the study. A PNCCP participant commented, “*I just felt the consent (form) was very long … what would be helpful would be …like they do for when you sign your escrow forms… to have somebody there to be transparent about what you are signing*”.

*Reduce requirement for, reduce the burden of, or be clear on data protection when asked to share medical records*. The PNCCP highlighted the requirement to share electronic health records for inclusion in the study as a potential barrier due to a history of medical mistrust, concern for maintaining privacy in their communities, and inconsistency in healthcare for some potential participants.

*Be flexible with time constraints*. The PNCCP also discussed the time commitment of the study as a barrier to participation. Specifically, the panelists discussed the barriers to participating in the three different 14-day in-house stays over 6 months component of the study. They discussed the economic burden of taking off work for a 14-day period. Additionally, the PNCCP discussed potential challenges with arranging child and elder care for an extended period of time. The panel discussed the potential emotional impact of being away from family members for a 14-day period. The PNCCP noted that making accommodations for family members to room together during the stay, participating in the 14-day stay alongside friends and/or family, and flexibility in the duration of the in-house stay would mitigate barriers to participation.

*Consider cultural preferences for bio-sampling*. Participants discussed concerns regarding the hair sample collection element of the study protocol to be used for the analysis of environmental chemicals and omics analysis. Particularly, the PNCCP highlighted the cultural differences related to the value of hair that might dissuade AA, as well as Native American, communities from participating in the study. A panel member shared, “*[The option to] opt out of hair/nails sampling is a good option. [I] was thinking about cultural humility and assumptions regarding this and some POC communities, especially African Americans*”.

*Promote diverse dietary preferences*. Similarly, panel members expressed concern that prescribed diet interventions might be inconsistent with cultural and/or personal preferences. Therefore, the preference was to offer adaptive dietary alternatives.

### 3.3. Study’s Response to PNCCP Recommendations

The feedback from the PNCCP was discussed by the research team to assess feasibility of implementation across the aforementioned four themes as detailed below:

*Reduce structural barriers to recruitment*. The PNCCP recommended explaining the clinical trial process and procedures, risks, and benefits concisely; a community outreach presentation was developed with PNCCP members’ help to provide a lay language summary of the clinical trial process, including study purpose and expectations, while giving community members a forum to ask questions about study procedures.

Study flexibility and accommodations were cited as a factor in participation. Following PNCCP recommendations, study visit hours were expanded to include weekends to accommodate those who worked a standard workday. Furthermore, the research team attended community outreach events equipped with tablets to allow potential participants to register for the study on-site, mitigating transportation barriers and improving geographic accessibility.

The panel members also recommended increasing compensation in this multi-site study to reflect the cost of living of the geographic area. However, the study compensation could not be modified as it was standardized across study sites nationally. To mitigate transportation costs, ride-share discounts were offered in addition to parking reimbursement.

*Need for recruitment materials to be culturally tailored and participant-centered.* The panel recommended making the research team more accessible; recruitment materials were modified to include multiple methods for research team contact (phone, email, website, QR code) over just the study website and QR code alone. This was particularly important as the need for digital literacy to navigate study registration was noted as a barrier to participation (and so direct connection to study staff was a proposed way to offset this barrier)

The study team removed scientific jargon and shortened messaging. The panel also recommended highlighting the short- and long-term benefits of participating in the study as a strategy to improve recruitment. Based on the recommendations from the panel, flyers were developed with recommended wording as well as imagery reflective of AA and Latino communities. However, as this is a national, multi-site study, even changes in local recruitment materials require review by the national study committee. On review by the NPH study oversight committee, some of the recommended modifications in scientific jargon, length of messaging, and changes to improve the readability of the flyers were not approved to maintain the branding and consistency of study materials. Dissemination of developed recruitment materials was restricted institutionally due to concern for competing interests with other ongoing clinical trials.

*Community-Engaged Trial Recruitment*. The PNCCP recommended partnering with community organizations to enhance the recruitment of diverse communities. The panel suggested some faith-based organizations and attending back-to-school nights, holiday events, and cultural food festivals in diverse communities. Additionally, they recommended outreach through partnerships with community diabetes and nutrition classes, local disease-based support groups (e.g., HIV support groups), and a weekly community fitness class. The study team created a calendar of PNCCP-identified outreach events. At these events, community members were encouraged to join the study as a group with their friends and family.

*Making nutrition trial procedures more inclusive and acceptable*. Diverse study staff were added to the research team so that individuals had the opportunity to go over the study process and consent with study staff in person to improve understanding and answer questions. This included bilingual individuals; institutional translation services were available as well.

The PNCCP recommended accommodating potential participants who choose not to provide access to electronic health records. However, the study protocol could not be modified for those unwilling to provide access to health records; eligibility criteria were standardized across study sites (study coordinators were instructed to emphasize to participants that their health records would be maintained securely to mitigate fear of loss of privacy).

The PNCCP also recommended allowing for flexibility in the duration of the 14-day in-house stay component of the study to facilitate recruitment and retention. However, as the intervention duration was standardized, these changes were not permissible. Due to budgetary limitations, child and elder care costs could not be reimbursed for study participants. Participants who worked remotely were permitted to continue to work during their in-house stay to mitigate the potential economic burden of missing work. Those needing child and/or elder care to allow for participation could not be accommodated (the study team’s request for expansion of the national budget to include this was denied). The only change to the protocol in this regard was to allow participants to have visitors during their in-house stay (but no overnight visits were permitted, and there was no ability to care for others at the study site).

The panelists recommended providing dietary alternatives to prescribed dietary interventions to accommodate for participants’ cultural and personal preferences. Dietary interventions were standardized across study sites to allow for comparison and thus were not modifiable. The panelists recommended clarifying to potential participants that providing a hair sample is an optional study procedure in order to avoid deterring concerned participants from participating; community presentations were modified to emphasize the reasoning for which procedures were not modifiable or optional. Furthermore, research staff were trained to emphasize which study procedures were optional or modifiable and which could not be modified during individual discussions with potential participants following our work with the PNCCP.

## 4. Discussion

In this study, we used the DCE approach to (1) identify barriers and facilitators to AA and Latino participation in nutrition clinical trials in LAC and (2) develop culturally tailored recruitment resources and strategies to improve the recruitment and retention of minority communities in randomized nutritional clinical trials in LAC. We consider some of the important findings and discussion points across the four major themes: (1) structural barriers to recruitment, (2) lack of culturally tailored recruitment materials, (3) inadequate community engagement, and (4) lack of flexibility in trial procedures to meet cultural considerations and socioeconomic burdens.

### 4.1. Structural Barriers to Recruitment

Similar to other studies, we found that lack of understanding and awareness of clinical trials was a barrier to participation amongst minority communities [29]. Lack of understanding of clinical trial procedures can further common misconceptions minority communities may have regarding clinical research [29,30], such as clinical trials using individuals as “guinea pigs”. Thus, improving understanding of clinical trials is key to increasing recruitment and retention of minority communities, as well as building trust.

Lack of clinical trial accessibility is another important barrier to clinical trial participation amongst AA and Latino communities. Similar to other studies, we identified transportation and the need for child-care as barriers to the participation of URM in clinical trials [31,32]. In one study, when AA adults were surveyed on barriers to participation in clinical trials the most common responses were time constraints (32%) followed by transportation (28%) [29]. Time burden is often tied to family obligations such as caretaking for children [31] or elderly family members. Rote et al. found that compared to non-Hispanic whites (NHWs), AAs and Latinos engage in more frequent caregiving of elders [33] and thus are more likely to have to consider caretaking responsibilities in their decision to participate in research.

The PNCCP identified inadequate compensation as a potential barrier to participation amongst AA and Latino communities. AA and Latino communities are more likely to be in poverty than NHW peers [34]. Thus, the financial burden from participating in clinical trials may be disproportionately greater amongst these communities. Several studies have reported that appropriate compensation may be more heavily weighed amongst minority communities [29,35]. Furthermore, our study findings highlighted the concern that compensation did not reflect the differences in the cost of living across study sites. The cost of living in LAC is approximately 50% higher than the national average. Thus, multi-site studies that set uniform compensation amounts nationally may actually undercompensate participants for their time. One important change is to consider compensation for research participants based on the cost of living for the region. Also noted was the concern that receiving compensation from participation in a clinical trial might impact taxes and governmental benefits received—these are important issues for research institutions to address when working with underserved populations.

### 4.2. Need for Recruitment Materials to Be Culturally Tailored and Participant-Centered

The importance of having figures that reflect a diverse community was highlighted as a facilitator to the recruitment of AA and Latino communities. Several studies have reported that potential participants are more likely to participate in a research study if there is racial concordance with research staff and that lack of diversity is a common barrier [31,36,37]. Recruitment materials with images reflective of an individual’s community improves the perceived inclusivity of the study, thus facilitating recruitment of diverse communities.

Furthermore, recruitment materials in the preferred language of potential participants and language concordant staff was noted as a potential facilitator to recruitment amongst Latino communities. Accommodations for those who speak languages other than English is particularly of importance in LAC, where 23% of individuals report speaking English less than very well and 37% of individuals report speaking Spanish at home [22], which is significantly higher than the national average. When clinical research coordinators were surveyed, 69% noted that having an in-person interpreter was more effective in recruitment of a potential participant than a phone interpreter [38]. Furthermore, studies have shown that when Spanish-speaking patients are seen by language-concordant providers, there is a more significant perception of trust [39]. Thus, recruitment materials and staff who are Spanish speakers may enhance recruitment in Latino communities, as well as helping to build trust with recruiting institutions.

The need for Internet access, Internet-connected devices, and digital literacy to facilitate study participation was highlighted by the PNCCP. The digital divide disproportionately affects low-income and minority patients [40], which certainly may introduce a greater impact on participation for URM populations. If we expect digital access or digital health to be a requirement or expectation of use in clinical trials, then we must develop management and outreach interventions that encompass digital device access, internet access, and digital literacy skills for prospective patients in a culturally and linguistically concordant manner. Such approaches could include things like digital health use screening as part of intake processes in clinical trials, digital health navigators to assist patients, and clinical trial programs to supply patients with free high-quality electronic devices and home internet [41,42].

The PNNCP results demonstrate the importance of highlighting study participation benefits in promoting recruitment, which has been described before. Several studies have reported that minority communities are more likely to participate in a clinical trial if it were to benefit them or their community [30,32,43]. Gadegbeku found that among AAs, personal health concerns and the potential of helping others were important motivating factors in the decision to participate in a clinical trial, with 52% rating it most important and 81% very important, respectively [44]. Highlighting how participating in clinical trial research may benefit the individual and their community has the potential to help to mitigate historical distrust regarding clinal trials amongst diverse communities.

#### 4.2.1. Community-Engaged Trial Recruitment

Similar to other studies, in both Latino and AA communities, mistrust of researchers and the health system was highlighted as a potential barrier to participation in clinical trials [35,45]. Williams et al. found that mistrust stemming from historical mistreatment such as in the Tuskegee Syphilis study still influenced AAs’ views on clinical research [32]. Furthermore, perceived fear that research will benefit NHWs or the research institution more than AA individuals also contributes to this mistrust [46]. AA and Latino communities have expressed fear of being used for experimental purposes as a barrier to participating in clinical trials [31]. Furthermore, fear of perceived racism and mistreatment experienced as a patient potentially being mirrored in clinical research has been noted to be a barrier to participation in clinical trials in minority communities. Thus, mistrust due to both historical tragedies and experienced institutional racism in healthcare impacts the decisions of AA and Latino communities’ participation in clinical trials. Still, some research studies indicate that amongst AA communities, both research participants and those who elect not to participate in research have similar mistrust of physicians [44]; thus, mistrust may not play as important a role in recruitment as once believed.

The panelists advised us to facilitate recruitment using community organizations and trusted community members, rather than physicians and research staff, to disseminate study information. Wood et al. found that compared to White adults, non-White adults were more likely to obtain information about clinical trials from other patients (24.5% versus 12.0%, *p* = 0.04) rather than from physicians or the Internet [47]. This highlights the value that minority communities place on receiving information regarding clinical trials from peers. Similar to other studies, the PNCCP noted that minority communities are more likely to participate in clinical trials when approached in trusted community institutions such as churches [32,48] and schools [32]. A focus group including 64 individuals aimed at identifying barriers and facilitators to the participation of AAs in clinical research found that while all individuals recommended recruiting from churches and clergy, less than 70% recommended recruiting from radio, social media, television, or flyers [35]. In the same study, more than 80% of participants noted that research staff who participants can identify with and trust could facilitate recruitment of AAs in clinical trials.

A key facilitator to recruitment shared by the panel was the importance of having diverse clinical staff who reflected the community. Racial concordance has been identified as a motivator for participation in clinical trials in both AA and Latino communities [31]. This is likely due to an increased sense of trust associated with racial or language concordance that has been noted in several clinical trials [36,49]. Workforce diversity of investigators and research teams should be an important factor in the development of future clinical trials.

#### 4.2.2. Making Nutrition Trial Procedures More Inclusive and Acceptable

Lack of understanding of the procedures and time required in a clinical trial has the potential of leaving participants feeling misinformed about the clinical trial process and thus worsening perceived mistrust amongst diverse communities. To improve understanding of the consent process, the PNCCP recommended limiting scientific language and making research staff available to go over the consent in detail with participants. Similarly, Quin et al. surveyed AA and Latino community members on strategies to enhance understanding of the informed consent process; they found the most helpful strategies reported were taking home the information, one-on-one discussion, and offering more than one meeting [50]. Additionally, changing the formatting of the consent to plain language was rated by approximately 80% of individuals as being very helpful in understanding the consent process.

An important potential barrier to many studies is the resistance to using both national and local recruitment materials, a position taken in many national clinical trials. Despite knowledge in almost all sectors of society that the local context is the most important, there remains an emphasis on the sanctity of recruitment strategies that not only reify structural barriers (e.g., racial and ethnic, socioeconomic, education and digital readiness) but also, by limiting participation solely through restrictive outreach strategies, actually lead to a major limitation on the generalizability of study findings.

Another key barrier to recruitment in our study is the lack of flexibility of the study protocol regarding time burden and procedures. The PNCCP noted that the length of time required to spend away from home during the study could have a significant social and financial consequence for potential participants. They recommended allowing family members to co-habitat during in-house stays. Also, noted was the lack of cultural considerations in the study procedures, specifically with the dietary interventions. The PNCCP noted that having culturally tailored diets could facilitate participation in a nutritional clinical trial and improve generalizability. However, this could not be implemented in our multi-site national clinical trial as the dietary interventions were standardized across sites. In order to incorporate cultural preferences into dietary interventions, it is necessary to engage communities for insight during protocol development to ensure diet acceptability amongst communities of interest in order to enhance acceptability and adherence amongst diverse communities.

Along the same lines, the need for consideration of cultural preferences for bio-sampling was highlighted as an impactful factor in the consideration of diverse communities participating in a clinical trial. Hair samples have been used in research to examine environmental exposures and particularly in nutrition clinical trials to examine mineral and vitamin levels. Hair samples have also been used to assess for substance abuse, and concerns of having coordination with police services to detect drug use or possible planting of false data to make an arrest are major concerns in URM communities. In addition, the value of hair can vary across cultures and thus should be taken into consideration in clinical trials. For example, among AA women, hair is given more priority over body image [51]. Thus, hair collection in clinical trials could discourage participation in research amongst diverse communities. In order to improve participation of AA communities in clinical trials that involve bio-sampling, specifically hair collection, establishing trust with community members through long-term engagement is essential. Furthermore, understanding and respecting the cultural significance of hair amongst AA communities is necessary to establish trust. Furthermore, providing materials that demonstrate in imagery formats can help mitigate fear around hair collection [52].

Similar to other studies, concern for inadequate protection of personal information was also noted to be a potential barrier to participation in clinical trials [32]. This is in part due to historical and experienced mistrust amongst diverse communities. Thus, participating in clinical trials that require access to electronic health records might dissuade diverse communities from participating. These are all important community and cultural factors that funding agencies, research institutions, and study teams must address as we work towards clinical trial inclusion of historically and contemporarily marginalized racial and ethnic minority populations.

### 4.3. Next Steps, Limitations

At the completion of the NPH study, the study team will evaluate the effectiveness of the culturally tailored engagement, recruitment, and retention strategies in encouraging clinical trial participation among minority adults from LAC, comparing the success of recruitment at UCLA with other LAC and national sites.

This work had several strengths. In previous studies, community-engaged research has been shown to increase recruitment and retention into clinical trials [15,53,54]. To our knowledge, this study is the first detailing the use of DCE to improve the recruitment and retention of AA and Latino communities in a randomized nutrition clinical trial. Using the DCE approach, we were able to elicit key barriers and facilitators to the recruitment and retention of diverse communities in nutrition clinical trials from a PNCCP representative of these communities. Recommendations from the panel’s discussions were implemented through modifications in study staff diversity and training, access to the research team, and outreach strategies (to the extent possible, given institutional and systemic barriers). Another impactful element of the study was the experience of the PNCCP members. Many of the panel members expressed that they felt their participation in the PNCCP would have a positive impact on their community’s experience in this clinical trial and feeling very satisfied with their experience participating in the PNCCP.

Our study had limitations. We were unable to incorporate all of the recommendations of the PNCCP due to limitations in staffing, resources, and restrictions on protocol modifications given multisite standard protocol across the US. Though not all the panel’s suggestions were implemented for this current work, these recommendations can inform future studies and point to the importance of involving a community consultant panel early in the protocol and recruitment strategy development phase of clinical trials. This work can also inform efforts to change policies, structures, and institutional workflows that can then better serve the goal of authentic clinical trial diversity.

The PNCCP only included English-speaking panelists, and the discussion sessions were only held in English. Lack of representation of Spanish-only speakers or Spanish speakers with limited English proficiency may limit the generalizability of the panel’s recommendations, specifically in LAC where nearly one-quarter of individuals report speaking English less than very well.

## 5. Conclusions

Improving the diversity of participants in nutrition clinical trials is essential to reducing the racial and ethnic disparities in CMD prevalence and outcomes. The PNCCP approach provides a framework for improving the understanding of recruitment and retention of diverse individuals in nutrition clinical trials, as well as developing culturally tailored recruitment strategies through community engagement which can be used to enhance the participation of diverse individuals in future nutrition clinical trials. This study provides important insight into the experiences of AA and Latino communities considering participation in nutrition clinical trials in LAC, and also a window into the real-time bureaucracies that potentially limit impactful change and study generalizability, and therefore must be addressed if we are to conduct the best science for all Americans.

## Figures and Tables

**Table 1:** PNCCP sessions curriculum presentation and discussion topics.

Session	PNPNCCP Session Agenda Topics
Meeting 1	Presentation Topics: Introduction of community members, clinical trial research team, and partnerships Introduction of diet-related disparities Overview of the NPH study Roles and expectations Discussion Topics: NPH study questions and concerns Recommendations for advertising and disseminating information about the NPH study to Black and Latino communities
Meeting 2	Presentation Topics: Common diets that are being studied through UCLA Nutrition Clinical Trial Overview of Modules 1 and 2 of the NPH study All of Us (AOU) research program Discussion Topics: Personal experiences with the diets being studied Recommendations on clinical trial compensation Potential barriers
Meeting 3	Presentation Topics: Importance of study recruitment and retention Discussion Topics: Discussion of national AoU recruitment materials (e.g., flyers, website) Q&A of potential questions potential participants may have about the clinical trial
Meeting 4	Presentation Topics: Current recruitment/enrollment website Review of survey results on NPH recruitment materials Discussion Topics: Discussion of potential benefits of participating in study Outreach strategies to Black/Latinx communities Feedback/response to AoU website recruitment materials
Meeting 5	Presentation Topics: Review of PNPNCCP survey results on barriers and/or facilitators to Black/Latino communities study participation Video and infographic overview and development process Discussion Topics: Recommendations on how to address the barriers highlighted in PNCCP survey Review and feedback on current recruitment videos for NPH study
Meeting 6	Presentation Topics: Elements of consent: description of study, risks and benefits, and compensation for Module 1 and 2 of the NPH study Clinical trial procedure for Module 1 and 2 Proposed compensation plan for trial participation Discussion Topics: Q&A on enrollment and procedure process of Module 1 and 2 Potential barriers among cultural communities to acceptability of trial procedures (e.g., hair sampling) Feedback on compensation methods
Meeting 7	Presentation Topics: Elements of consent: description of study, risks and benefits, and compensation for Module 3 Recap of outreach suggestions for study recruitment Discussion Topics: Potential organizations for outreach events Q&A on inclusion/exclusion process and consenting process Questions on Module 3 trial procedures
Meeting 8	Presentation Topics: Review of AoU website Overview of participant retention and its importance as well as common reasons for poor retention Discussion Topics: Potential retention strategies that would be successful for amongst diverse communities
Meeting 9	Presentation Topics: Updates on outreach events attended Review of AoU materials available in English and Spanish Discussion Topics: Feedback on AoU materials Community events for outreach recommended by PNCCP
Meeting 10	Presentation Topics: Outreach strategy review Calendar of events review Review of community outreach presentation Discussion Topics: Feedback on community outreach presentation Community events for outreach recommended by PNCCP
Meeting 11	Presentation Topics: Update on outreach events attended Summary of the recommendations PNCCP provided and their implementation Participant experience survey results Discussion Topics: Discussion on experience as PNCCP member Review of modified recruitment materials Community events for outreach recommended by PNCCP

**Table 2:** Demographics and experience of the PNCCP (N = 17).

Demographics	N (%)
Gender	
Female	8 (47.1)
Male	8 (47.1)
Prefer not to answer	1 (5.9)
Age group	
18–35	4 (23.5)
36–49	6 (35.2)
50–65	3 (17.6)
>65	4 (23.5)
Education	
High School or GED	2 (11.8)
Associate’s or Technical Degree	1 (6.2)
Bachelor’s Degree	5 (29.4)
Postgraduate (Master’s, PhD, etc.)	9 (52.9)
Race/ethnicity (check all that apply)	
Black/African American	6 (35.3)
Black/African American, White	1 (5.9)
Filipino/Filipina	1 (5.9)
Latino/Latina	8 (47.1)
White/Caucasian	1 (5.9)
Immigration status	
First generation	5 (29.4)
Second generation	4 (23.5)
Third generation	4 (23.5)
Non-immigrant	4 (23.5)
Groups identified with (check all that apply)	
Chronic disease	4 (23.5)
Disability	1 (6.2)
Experience with homelessness	2 (11.8)
Experience with mental health	2 (11.8)
Experience with substance abuse	2 (11.8)
LGBTQ+	6 (35.2)
Lower income	4 (23.5)
Receiver of public benefits	3 (17.6)
Veterans	5 (29.4)
Other	6 (35.2)
Have nutrition-related chronic disease	
Diabetes	2 (11.8)
High blood pressure	4 (23.5)
High cholesterol	2 (11.8)
Other	3 (17.6)
None	7 (41.2)
Prefer not to answer	2 (11.8)
Prior research experience	
Previously served on a CCP	7 (41.2)
Worked on a research study with an academic institution	11(64.7)

**Table 3:** PNCCP recommendations.

Themes	Recommendations	Implementation
Reduce structural barriers to recruitment		
*Clinical trial understanding*	Educate potential participants about important concepts and terminology around clinical trials.	Presentations were offered to the community detailing the clinical trial process including risks and benefits.
*Clinical trial accessibility*	Improve study flexibility to ease participation.	On-site study registration offered at local community events.
Ability to secure care for a child or family member	Accommodations are needed for participant buy-in.	Due to budgetary limitations, child and/or elder care costs could not be reimbursed. Study visit hours were expanded to include weekends.
*Compensation considerations*		
Inadequate compensation for cost of living in the region	Monetary compensation should be fair with competitive pay for the geographic area.	Study compensation could not be modified as it was standardized across study sites. Ride-share discounts were offered
Monetary compensation may impact taxes and benefits		
Culturally tailored and participant-centered recruitment materials		
*Reduce scientific jargon on recruitment materials*	Reduce scientific language	Flyers were developed with recommended wording and imagery reflective of AA and Latino communities, as well as the addition of study benefits to the individual and society; however, they were not approved by the study oversight committee
*Simplify messaging*	Be concise in messaging with the goal of immediately capturing audience’s attention	
*Advertise and highlight study participation benefits*	Highlight short- and long-term benefits of participation to participants.	
*Increase representation in images*	Include additional underrepresented groups reflective of the community prominently in materials.	Separate flyers were developed to target the AA and Latino communities with images reflective of the respective communities.
	Develop separate materials targeted to Latino and AA communities.	Separate flyers created targeting Latino and AA communities. Flyers targeting the Latino community were translated into Spanish.
*Improve the diversity of contact methods*	Improve accessibility to research team.	Additional methods of contacting the research team provided (phone, email, website, QR code).
Community engaged trial recruitment		
*Community partnered outreach*		
Partner with community-based organizations	Use preexisting communities (e.g., schools, faith based organizations) and social networks for recruitment.	The PNCCP identified outreach events to attend (e.g., fitness events, taste of soul festival) and organizations with which to partner.
*Utilize networking*		Community encouraged to join study with friends and family.
*Improve study staff/researcher representation*	Study staff should be reflective of the diverse community at outreach events.	Diverse study staff were added to the study team.
Make nutrition trial procedures more inclusive and acceptable		
*Simplify consent form*		Images were used to explain complicated procedures. Text could not be modified as it was standardized across study sites.
*Reduce requirement to share medical records*		
Fear of losing privacy sharing electronic health records	Study should accommodate those who opt out of sharing medical records.	The study protocol did allow for modifications as it was standardized across study sites.
*Be flexible with time requirements*	Time commitment of components of the study (14-day hotel stay) may be burdensome to participants. Advise flexibility in duration of stay.	
*Promote diverse dietary preferences*	Dietary alternatives should be provided to accommodate for cultural and personal preferences.	
*Consider cultural preferences for bio-sampling*		
Cultural concerns regarding hair sample collection		Presentations were modified to explain purpose of procedure and to emphasize that hair sample collection was optional.

## Data Availability

The data presented in this study (discussion transcripts) may be available on request from the corresponding author, but with select availability due to confidentiality of PNCCP discussions.
